# Cold Atmospheric Pressure Plasma May Prevent Oral Mucositis-Related Candidemia in Chemotherapy-Treated Rats

**DOI:** 10.3390/ijms252111496

**Published:** 2024-10-26

**Authors:** Aline da Graça Sampaio, Noala Vicensoto Moreira Milhan, Fellype do Nascimento, Konstantin Georgiev Kostov, Cristiane Yumi Koga-Ito

**Affiliations:** 1 Oral Health Applied Science Program, Institute of Science and Technology, São Paulo State University (UNESP), São José dos Campos 12245-000, SP, Brazil; alinnsampaio@gmail.com (A.d.G.S.); milhan.noala@gmail.com (N.V.M.M.); 2 Faculty of Engineering in Guaratinguetá, São Paulo State University (UNESP), Guaratinguetá 12516-410, SP, Brazilkonstantin.kostov@unesp.br (K.G.K.); 3 Department of Environment Engineering, Institute of Science and Technology, São Paulo State University (UNESP), São José dos Campos 12247-016, SP, Brazil

**Keywords:** mucositis, fungemia, cold atmospheric pressure plasma jets, plasma gases

## Abstract

Oral mucositis associated with candidiasis can causes systemic candidemia, posing a risk to cancer patients administered antineoplastic therapy. Cold atmospheric pressure plasma jets (CAPPJs) have antifungal and anti-inflammatory properties. This study evaluated the effects CAPPJs in preventing systemic fungal dissemination in a murine model of oral mucositis associated with candidiasis. Forty Wistar rats were divided into groups: CAPPJs (treated) and non-treated controls (for comparison), with subgroups subject to 24 and 72 h of treatment (*n* = 10 each). Four cycles of chemotherapy (cisplatin and 5-fluorouracil (5-FU)) were administered, followed by oral inoculation of *Candida albicans* for 3 days. Mucosal damage was induced on the lateral side of tongue with 50% acetic acid. CAPPJ treatment was performed on the lesion for 5 min (2 days). Body weight was assessed daily. Fungal dissemination was conducted using organ macerates and plated on Sabouraud Agar with chloramphenicol. Blood samples were obtained for blood count tests. Chemotherapy affected the general health of the animals, as evidenced by body weight loss. Treatment with CAPPJs showed an inhibitory effect on *C. albicans*, with a significant reduction in fungal recovery from the tongue after 24 h (*p* < 0.05). Interestingly, systemic fungal dissemination was significantly reduced after 24 and 72 h of treatment when compared to control (*p* < 0.05). Taken together, these results suggest that CAPPJs have potential for clinical application in patients with oral mucositis at risk of candidemia.

## 1. Introduction

Chemotherapy-induced oropharyngeal mucositis is a common complication of antineoplastic treatment that can negatively impact its success [1], as it is related to high morbidity [2]. Mucositis is considered a side effect generally caused by chemotherapy’s nonspecific action on proliferative tissues. As a result, healthy cells with high turnover, such as oral epithelial cells, are commonly affected [1,3,4,5,6,7,8,9]. During the process, clinical oral lesions that can be seen as erythema and/or ulcers occur as a result of tissue damage caused by the harmful effects of reactive species generated by the chemotherapy drug. Additionally, oral infections can occur as a secondary effect due to the immunosuppressive action of cancer treatment [10,11,12]. This effect of direct toxicity by chemotherapy drugs on tissues is known as direct stomatotoxicity and can affect several oral regions, such as the tongue, the soft palate, the pharyngeal area [1,12], jugal mucosa, the floor of the mouth, and the lips [10,13].

A systematic review of oncological protocols reported that platinum derivatives (cisplatin and oxaliplatin) and alkylated (5-fluorouracil, 5-FU) chemotherapy agents are frequently associated with oral mucositis [14]. Around 20–80% of cancer patients with tumors in the head and neck region undergoing chemotherapy develop oral mucositis, and the incidence rate can vary according to the dose and pharmacological agent administered [14,15,16,17]. Prolonged treatments can increase the severity of the mucositis [17]. It was described that 1/3 of patients with mucositis reported difficulty speaking, 90% have difficulty ingesting water and food, and 85% have weight loss [18,19]. Additionally, they usually present poor oral hygiene, increasing the risk of infection [20,21].

Despite the high frequency of mucositis and severe local and systemic consequences, the lack of effective interventions has been frustrating for patients and caregivers [1]. There are many studies focused on preventing and treating mucositis; however, there is still no consensus protocol for prophylaxis and treatment of this condition [5]. Primary oral care such as tooth brushing, use of dental floss, and mouth rinsing with chlorhexidine solution are considered only palliative measures for oral mucositis as they show limitations for reducing the severity of the lesions [19,22,23]. Other therapeutic interventions also have been also proposed. Treatments with honey, glutamine and vitamin E [24], herbal medications, and cryotherapy [17,25] presented good results, but deeper clinical investigations are still needed. Photo biomodulators show good clinical results; however, the use of different parameters and the unavailability of limiting dosimetry represent limitations to establishing a standardized protocol [26].

The oral cavity has a vast microbiome [27], and *Candida* spp. are commonly found [28,29,30]. *C. albicans* is the most prevalent among *Candida* spp., colonizing more than 80% of population [31]. Despite its commensality, *C. albicans* is an opportunistic pathogen that can proliferate and cause disease in the presence of local and/or systemic predisposing factors [31,32], such as immunosuppression due to cancer and chemotherapy. The incidence of oral lesions, such as oral candidiasis during chemotherapy can vary from 20 to 40% [33,34] and can be caused by *C. albicans* and non-*albicans* species [33,34,35].

Systemic infection, in turn, can be caused by the entry of pathogens through ulcerated oral mucositis in immunocompromised patients [5,36]. In these cases, *Candida* spp. can express invasive virulence factors [37] that contribute to fungemia. An in vivo study conducted by Katagiri et al. [38] showed the correlation among oral mucositis, oral candidiasis, and systemic fungal dissemination in a model where oral mucositis and candidiasis were simultaneously induced in mice. This study draws attention to the severity of this clinical condition, which goes beyond local occurrences and has the potential to cause sepsis.

New treatments with antimicrobial and anti-inflammatory potentialities are welcome for immunocompromised patients with both oral mucositis and oral candidiasis, as well as other kinds of oral lesions. In this context, cold atmospheric pressure plasma jets (CAPPJs) appears as a promising technology, due to its antimicrobial and anti-inflammatory effects. CAPPJs are composed of electrons, ions, and neutral particles, coming from the gas used during plasma production [39,40,41,42]. Reactive oxygen and nitrogen species (RONS) are particularly associated with the effects of plasma. Positive findings have been obtained for the treatment of infectious conditions, wound healing, inflammatory skin pathologies and neoplasia, with several applications in medicine and dentistry [43,44,45,46]. 

Previous studies have shown positive results for treating superficial infections caused by *Candida* spp. [47,48,49,50]. In a in vivo study, helium plasma inhibited the tissue invasion by *C. albicans*, with a low occurrence of inflammatory changes [51]. Similarly, an antifungal effect without damage to the superficial epithelium was reported in BALB/c mice with candidiasis treated with plasma helium mixed with oxygen (He/O_2_) for 4 min [52]. The prolonged use of plasma He/O_2_ for 10 min also showed no mucosal irritation in rabbits [53].

Considering the occurrence of refractory cases of oral candidiasis and the possibility of developing fungemia when it occurs in concomitance with oral mucositis lesions, this study aimed to evaluate whether CAPPJs can reduce the occurrence of *C. albicans* systemic dissemination in an in vivo model of chemotherapy-induced oral mucositis and candidiasis.

## 2. Results

### 2.1. Follow-Up of the Experiment: Validation of the Protocol

The protocol was validated by the establishment of oral candidiasis and mucositis lesions, in addition to the expected alterations in blood counts.

**Figure 1:**
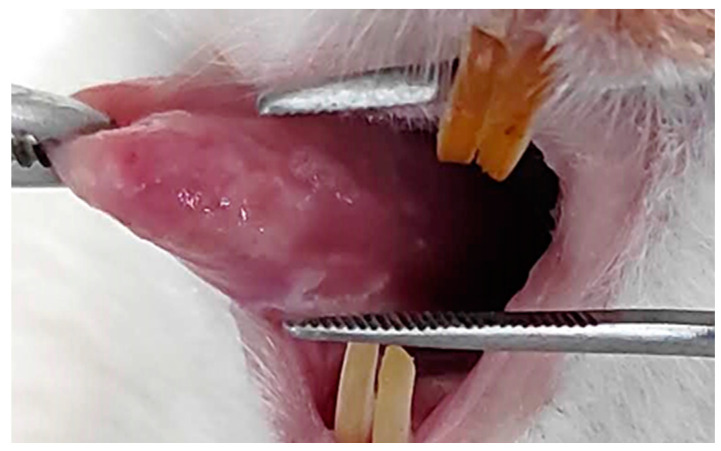
Image of an animal’s tongue with lesions on the lateral and ventral surface.

Clinical examination of the animals’ tongues revealed whitish patches and plaques on the dorsal, ventral, and lateral surfaces of the tongue (candidiasis), as well as erythematous and ulcerated areas on the lateral surfaces (oral mucositis; Figure 1). At the sites of oral mucositis, overlapping lesions were observed.

The successful establishment of immunosuppression was also detected by alterations in the red blood cell (RBC) and white blood cell (WBC) counts in blood collected on the day of the euthanasia. The results are presented in Table 1. There was reduction in the numbers of erythrocytes (RBCs) and mostly leukocytes (WBCs) in both groups at 24 h. Platelet levels were normal in the animals of the CAPPJs group euthanatized at 24 h and in both groups euthanatized at 72 h.

### 2.2. The CAPPJs Group Lost Less Weight After Treatment

**Figure 2:**
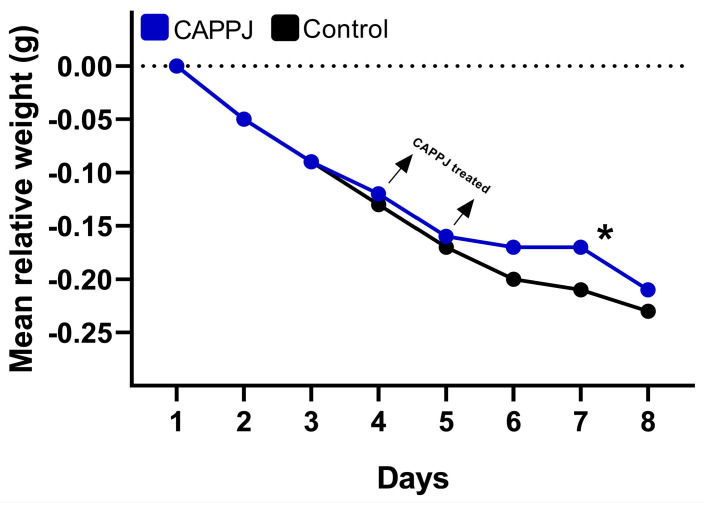
Mean relative weight of the animals under chemotherapy treatment with oral mucositis lesion associated with fungal contamination. The blue line represents the group treated with cold atmospheric pressure plasma jets (CAPPJs), and the black line represents the non-treated control. Dashed line represents axis *X*. The arrows indicate the days of CAPPJs treatment. * *p* < 0.05 (Student’s paired *t* test).

All the animals showed reduction in weight during the experiment (Figure 2) after the second day of chemotherapy. The mean weight loss varied between 385.1 ± 29.0 g and 407.7 ± 46.9 g (1st day) for the control and CAPPJs groups, respectively. On the 8th day, the mean weight loss in the control and treatment groups were 295.9 ± 33.7 and 322.1 ± 37.7 g, respectively. The CAPPJs group showed a tendency to lose less weight after treatment, with a significant difference in mean relative weight loss between groups (*p* = 0.036) at 7 days.

### 2.3. CAPPJs Reduced Fungal Systemic Dissemination

The number of animals positive for yeasts in each organ is presented in Table 2. Yeasts were isolated from all animals tongues, independent of the group and period analyzed. 

After 24 h, yeasts were recovered from 90% of the lungs as well as 50% of livers, spleens, and kidneys of the animals in the non-treated group. In the group treated with CAPPJs, yeasts were recovered only from 11.11% of the lungs and 22.22% of the kidneys.

Among the animals euthanatized 72 h after the CAPPJ treatment, yeasts were not recovered from the organs. Differently, for the control group at the same period of analysis, yeasts were recovered from 55.55% of the lungs, 44.44% of the livers, and 33.33% of the spleens. All the blood cultures were negative for yeasts.

There was a significant association between the use of CAPPJ treatment and the number of animals that presented a positive culture for *Candida* yeasts in the organs. The application of plasma reduced the systemic dissemination to the lung at both time periods and also to the liver and spleen 24 h after the last treatment with CAPPJs (*p* < 0.05; Table 2).

**Figure 3:**
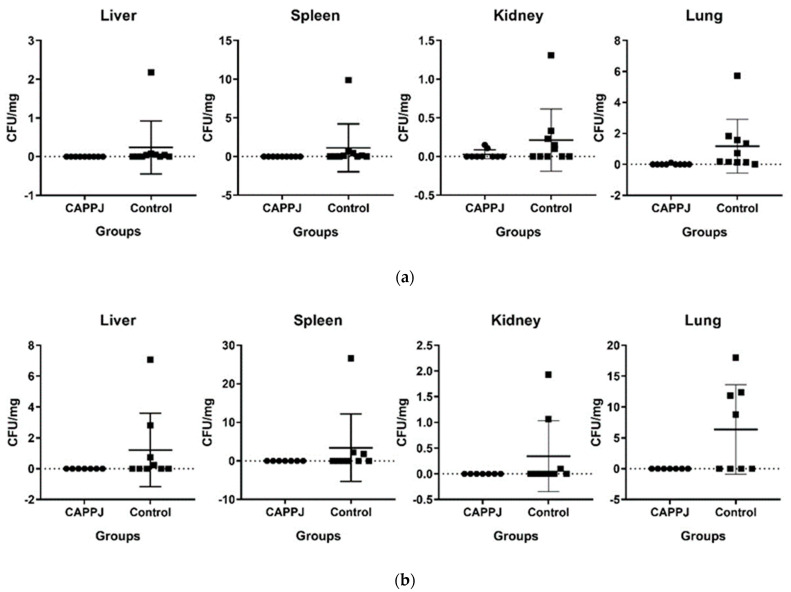
Number of viable fungal cells in the organs recovered (**a**) 24 and (**b**) 72 h after cold atmospheric pressure plasma jet treatment compared with the non-treated control group (non-treated). Values of mean and mean standard error (mean ± SEM) are represented.

Figure 3 shows the quantification of the fungal dissemination in the organs over time. Low fungal counts with tendency of 0 CFU/mg for the CAPPJs-treated group was observed after 24 and 72 h. On the contrary, higher fungal counts were observed in the control group in both periods.

**Figure 4:**
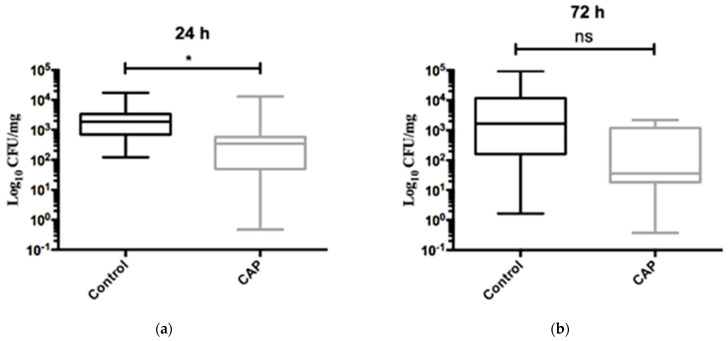
Counts of viable fungal cells (CFU/mg) recovered from the animals’ tongues 24 h (**a**) and 72 h (**b**) after the second session of plasma jet treatment. * *p* < 0.05. ns—non-significant (*p* > 0.05). (Mann–Whitney Test).

The number of viable fungal cells was also quantified in the tongue samples, and the results are shown in Figure 4. Significant reductions in yeast counts were detected 24 h after the second session of treatment with CAPPJs (*p* = 0.01). At 24 h after treatment, the mean values of CFU/mg organ were 3.32 × 10^3^ ± 5.01 × 10^3^ and 1.68 × 10^3^ ± 4.23 × 10^3^ for the control and treated groups, respectively. At 72 h after treatment, the mean values of the yeast counts were 1.35 × 10^4^ ± 3.08 × 10^4^ CFU/mg for the control group and 5.94 × 10^2^ ± 8.35 × 10^2^ CFU/mg) for CAPPJ treatment, which showed a tendency of fungal reduction (*p* > 0.05).

## 3. Discussion

The use of radiotherapy or chemotherapy for the treatment of head and neck cancers commonly leads to oral mucositis as a side effect, causing slight to severe damage to the buccal mucosa [5]. Interestingly, *Candida* spp. colonization is a risk factor for severe oral mucositis in these patients [55]. Severe cases of mucositis, in turn, exhibit ulcerations that can facilitate the propagation of this fungi into bloodstream causing disseminated fungal infection [56,57], as previously demonstrated in mice [38]. To our current knowledge, this is the first in vivo study that investigated the use of helium cold atmospheric pressure plasma jets (He-CAPPJs) as a tool to avoid candidemia in immunosuppressed animals with oral mucositis. 

Before the in vivo assessment, we confirmed in vitro that the CAPPJ parameters used in this study exhibit anti-*Candida* activity and low toxicity to oral cells when produced at power of 1 W, distance of 1.5 cm, and exposure time of 5 min [58]. Previous studies with *C. albicans* have also demonstrated the inhibitory and safety effect generated by helium-CAPPJs [27,49,50] at the same time and exposure distance [27,49]. In vivo plasma treatment equipment was previously analyzed and identified several reactive oxygen and nitrogen species (RONS) such as hydroxyl (OH), nitrogen oxide (NO), molecular nitrogen ions (N_2_^+^), oxygen (O), nitrogen (N), ozone (O_3_) and nitrogen dioxide (NO_2_) [58]. There are short or long-lived species that exhibit antimicrobial and anti-inflammatory characteristics [59]. In plasma research, the electric field and RONS produced by plasma on microbial cells can act on the cell wall or plasma membrane of microorganisms by interacting and altering their molecular bonds, resulting in a reduction in cellular surface tension that subsequently increases cellular pressure, the leakage of cytoplasmic content, cell rupture, and damage to DNA, resulting in microbial cell death [59,60,61,62]. As result of the interaction between plasma and mammalian cells, these biomolecules demonstrate an important antimicrobial tissue effect without causing cellular damage, side effects, or toxicity to the mammalian organism, thus reducing inflammation and accelerating tissue repair [59,63,64,65]. The reasons for the selectivity, i.e., cytotoxicity only to microbial cells, are the biochemical, metabolic, and cell cycle differences as well as the ratio between the surface area and volume of the cell [59,66].

The methodology for inducing mucositis and oral candidiasis, partially based on Kataguiri et al. [38], was also effective in this study. It was validated based on the clinical examinations and the hematological findings. Erythematous surfaces and ulcers covered by pseudomembranes that clinically characterize oral mucositis were observed [67]. Additionally, whitish patches and plaques indicated the presence of oral candidiasis [38,68]. Immunosuppressive conditions caused by the drugs usually modify hematological values [2,36,38]. In the literature, changes in the counts of red blood cell (RBCs), lymphocytes, neutrophils, and monocytes as well as hemoglobin levels have been demonstrated in association with chemotherapy, oral mucositis, and infections [2,69,70]. Cancer patients with candidemia usually show leukopenia, which can be considered a risk factor for infection [71,72]. In our study, the combination of cisplatin and 5-FU led to immunosuppression, resulting in a reduction in leukocytes (WBCs) and neutrophils [73]. A study conducted by Saftescu et al. [70] in humans that used 5-FU and cisplatin also reported slight anemia and an increased effect after treatment with cisplatin. In the present study, hematological alterations, including a reduction in RBCs and mostly WBCs, were observed, confirming the immunosuppression.

In our study, the animals of both groups showed weight loss. These results are in accordance with previous studies where chemotherapy treatment with 5-FU and cisplatin was used for the induction of oral mucositis caused weight loss [38,68]. The reduction of weight is a common and worrying clinical sign in cancer patients, associated with several adverse side effects, including diarrhea [74,75], which can lead to hospitalization, discontinuation of treatment, and potentially death. In agreement, in our study, symptoms of moderate diarrhea were observed in the animals of both groups, during and after chemotherapy cycles. Previous findings have demonstrated a relationship among weight loss, chemotherapy, the global health of the host, and the occurrence of mucositis [38,68]. In this study, there was a reduction of weight over time in both groups; however, the CAPPJs group presented a lower loss of weight after the treatment, which was significant on the 7th day. This finding can indicate an improvement in the general health of the treated group. Furthermore, CAPPJ treatment can contribute to antimicrobial and anti-inflammatory effects [51,59,76]. In agreement with our results, a previous work that used a similar methodology observed prominent weight loss in non-treated animals compared with the treated ones [38]. 

It is important to highlight that the occurrence of infectious disease, such as candidiasis, requires a complex microorganism–host interaction, involving both the expression of virulence factors generated by *Candida* and the interactions of the microflora and the host’s immune system [77]. In cases of ulcerated mucositis, the opportunistic behavior of this pathogen can lead to systemic dissemination [38]. In this study, there was fungal dissemination of *C. albicans* to the organs, similar to that detected by Katagiri et al. [38] and Ninomiya et al. [68].

Our study investigated the presence of fungi in the kidney, liver, lung, and spleen. The non-treated group presented higher fungal counts in the lungs followed by the spleen in both periods of euthanasia (24 h and 72 h). Katagiri et al. [38] also detected fungal dissemination 24 h after the last procedure. However, differently from our study, Katagiri et al. [38] examined only the kidney and liver in non-treated animals, and a higher fungal concentration was found in the liver. According to previous works, the dissemination first occurs into the liver and spleen under immunosuppressive conditions [78,79]. A study with mice observed that neutropenia and disruption of the gastrointestinal mucosa are mandatory for fungal dissemination, which was not seen only under conditions of neutropenia. In the present study, there was neutropenia and disruption of oral mucosa due to oral mucositis, which probably contributed to fungal dissemination following the same mechanism observed in cases of gastrointestinal disruption [79].

In this study, CAPPJs were applied to oral mucositis lesions in the tongue of rats for 5 min over 2 consecutive days to evaluate the reduction in oral candidiasis and consequently fungal dissemination. In addition to the clinical signs of oral candidiasis, fungus was present on the tongues of all the animals, as previously reported by authors that followed the same methodology [38]. CAPPJ treatment showed significant fungal reduction in the tongues of the animals after 24 h of plasma exposure, although a tendency of fungal reduction was also observed after 72 h.

As a consequence, significant differences between control and treated groups were detected concerning fungal dissemination. The CAPPJs group showed low variability in fungal recovery, with a tendency toward complete fungal clearance. Ninomiya et al. [68] used similar methodology to treat the tongues of mice with antifungal drugs (2% miconazole gel administered orally at 60 mg/kg and 1 mg/mL fluconazole administered intravenously) for 3 days. They observed a reduction in white patches on the tongue and a reduction in *C. albicans* dissemination to the organs. The animals treated with fluconazole presented greater fungal reduction than the group treated with miconazole. As far as we know, this previous study with antifungal agents was the only that reproduced and treated a model of chemotherapy-induced mucositis and candidiasis. Therefore, a comparation of CAPPJs with other kinds of similar therapies to avoid candidemia is not currently possible, which reinforces the innovative character of our study.

Positive blood cultures were not detected in our study. One hypothesis for this finding can be the low fungal presence and insufficient fungal recovery from the blood. It is worth emphasizing that previous studies have shown that positive blood cultures occur in less than 50% of the samples analyzed [80,81], delaying the diagnosis [82], which can be lethal for the patient. Previous studies by Katagiri et al. [38] and Ninomiya et al. [68] observed a fungal presence in samples of blood in 60% of the non-treated animals and 40% of the ones treated with oral care. However, in these studies, the experimental infection was induced using inoculation with a fungal suspension, which can cause an enhanced fungal presence in other organs via the respiratory and gastrointestinal tracts, consequently causing higher fungal dissemination. A pilot test carried out by our group showed that the inoculation of a fungal suspension into the rats’ mouths lead to a high rate of early mortality. Therefore, the induction of oral candidiasis in our work was performed with the aid of a swab previously soaked in a fungal suspension, which was also successfully employed by Borges et al. in 2018 [51] and Sampaio et al. in 2021 [83].

Taken together, our innovative findings suggest that treatment with CAPPJs was effective to reduce systemic fungal dissemination in rats administered chemotherapy, which can indicate a significant impact on the prevention of candidemia. These results are promising and suggest that CAPPJs can become an effective adjuvant therapy for cancer patients with the simultaneous occurrence of oral mucositis and candidiasis induced by immunosuppression during cancer treatment. Futures studies are required to understand whether this action is related only to the antimicrobial potential of CAPPJs against *C. albicans* or if there is also a reduction in local microcirculation due to an anti-inflammatory action in oral mucositis areas.

## 4. Materials and Methods

### 4.1. Plasma Source

The physical parameters of the cold atmospheric pressure plasma jet (CAPPJ) device employed in this study, as well as its most effective biological conditions of distance and time of exposure were previously described by Nascimento et al. [58]. The electrical characteristics of the device was developed following the DINSpec guidelines (safety, low risk, and efficacy of the medical plasma source). The device basically consists of a portable power supply and a dielectric barrier discharge (DBD) reactor connected to a flexible plastic tube with a voltage up to 20 kV, frequency of 60 Hz, and power of 1 W. The device uses helium gas (He 99% purity; Air Liquide, São Paulo, SP, Brazil) with 2.0 ± 0.01 slm (standard liter per minute) controlled flow rate (OMEGA, model FMA5518A) and operates at a distance of 1.5 cm between the plasma outlet and target. Under these operating conditions, the patient leakage current is lower than 100 µA-AC, the gas temperature is of the order of 33 °C, and the target temperature does not exceed 30 °C [58]. Optical emission spectroscopy revealed the production of RONS like hydroxyl (OH), nitrogen oxide (NO), molecular nitrogen ions (N_2_^+^), and atomic oxygen (O) and nitrogen (N). In addition, when employing gas detectors to quantify the production of ozone (O_3_), nitrogen dioxide (NO_2_), and NO, it was found that the device produces up to 0.05 ppm of O_3_, 0.02 ppm of NO_2_, and 0.01 ppm of NO, with the plasma jet impinging on a conducting target [58]. These physical parameters demonstrated antifungal activity without cytotoxic to oral cells [58].

### 4.2. Fungal Culture

*Candida albicans* (ATCC 18804) stored in Sabouraud dextrose broth (SD) containing 20% glycerol at −80 °C was reactivated in fresh Sabouraud dextrose agar. The plates were incubated at 37 °C for 24 h under aerobic condition. Subsequently, a standardized fungal suspension containing 10^8^ cells/mL was prepared in sterile saline solution (0.9% NaCl) using a spectrophotometer (λ = 530 nm and O.D.= 1.258; B582, Micronal, São Paulo, SP, Brazil).

### 4.3. Animals

The Ethics Committee on the Use of Animals approved the experimental protocol of this study (process # 16/2019). All the experiments were conducted in accordance with the ARRIVE (Animal Research: Reporting of In Vivo Experiments) guidelines. A total of 40 male rats (*Rathus norvegicus*), aged between 90 and 100 days, were maintained under controlled conditions of temperature (22 °C) and 12 h day–night cycles. Rats were housed in ventilated racks (Alesco, Brazil) with free access to water and food. Prior to the experiments, the animals received topical antiparasitic medication (1 drop/animal; Revolution 6%—Zoetis, SP, Brazil) and a single oral dose of dewormer (1 mL/kg; Vermotrix, Lema biologic, Ponte Nova, MG, Brazil). To prevent microbial contamination, the test was conducted in a sterile environment, including the materials provided to the animals (food, water, wood shavings, bottles, and jars) and isolators previously sanitized with alcohol 70%. To avoid bacterial infection, tetracycline (0.83 g/L) was administered in water according Borges et al. [51]. The animals were divided randomly in two major (1 and 2) groups and four subgroups (A–D), according to the treatment and time of euthanasia, respectively. (1) The control or non-treated group included animals with oral mucositis and oral candidiasis that were evaluated after (A) 24 and (B) 72 h. (2) The treated group (CAPPJs group) included animals with oral mucositis and oral candidiasis treated with CAPPJs that were evaluated after 24 (C) and 72 h (D).

### 4.4. Experimental Procedure

**Figure 5:**
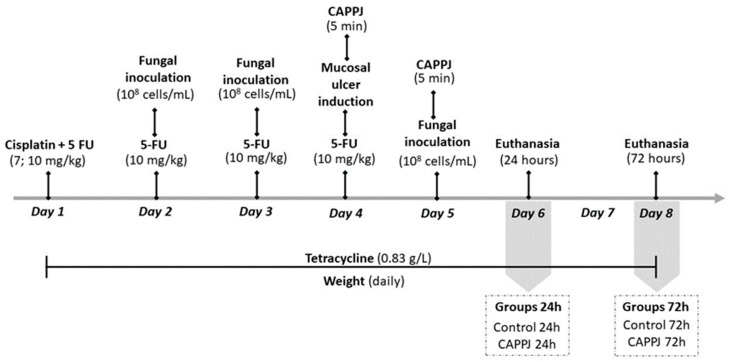
Experimental timeline with all the stages of the experiment until euthanasia.

The methodologies to induce immunosuppression by chemotherapy, to delimit oral mucositis lesions, and to establish oral candidiasis were based on Katagiri et al. [38], with some modifications. The chemotherapy treatment was administered intraperitoneally as a single dose of 7 mg/kg cisplatin (Libbs, Embu das Artes, SP, Brazil) on day 1, and 10 mg/kg of 5-fluorouracil (5-FU, Libbs, Embu das Artes, SP, Brazil) for was administered for 4 consecutive days (Figure 5). To perform the experimental procedures, the animals were anesthetized with ketamine (95 mg/kg, Ceva, Paulínia, SP, Brazil) and xylazine (10 mg/kg, Ceva, Paulínia, SP, Brazil). To induce oral candidiasis, fungal inoculations were performed on the 2nd, 3rd and 5th days. For this, sterile swabs were soaked in the standardized fungal suspension for 5 min. Then, *C. albicans* suspension were inoculated in two stages. First, the suspension was inoculated in all regions of the oral cavity for 15 s and, the swab was maintained in the dorsal area of the tongue for 5 min, according to Borges et al. [51] and Sampaio et al. [83]. The delimitation of mucositis was performed on the 4th day of the experiment. For this purpose, a sterile swab moistened with acetic acid solution (50%) [84,85] was applied to the lateral region of the tongue for 60 s on each side, followed by washing with a swab moistened with physiologic solution (Ever care, Nova Odessa, SP, Brazil).

**Figure 6:**
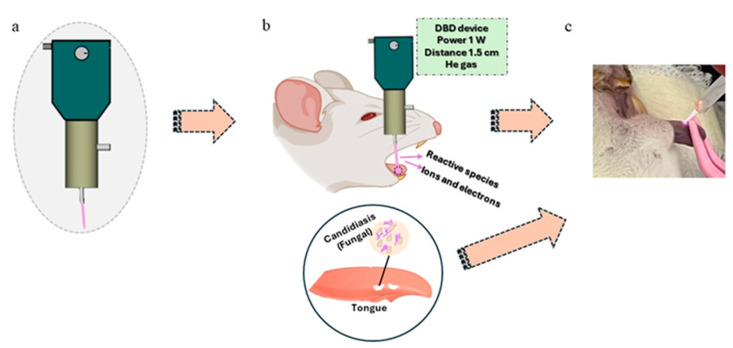
Schematization of the treatment with cold atmospheric pressure plasma jets (CAPPJs) (**a**,**b**) and (**c**) clinical exposure of CAPPJs to the lesions located on the tongue.

The treatment with CAPPJs was performed on the 4th day after delimiting the mucositis lesion and on the 5th day after fungal inoculation. For treatment, the animals were anesthetized as described before. Prior to CAPPJ exposure, the tongue was humidified with one drop of physiologic solution to avoid dryness during the treatment. Then, the CAPPJs were applied in the central area of the lateral border of the tongue for 5 min (Figure 6). On the 6th and 8th days, prior to euthanasia, the animals were anesthetized, and blood samples were collected by cardiac puncture for hemogram analysis. Then, to investigate the occurrence of systemic infection, a triple dose of anesthetic was administered. After euthanasia, the organs (lung, spleen, liver, kidney, and tongue) were collected for microbiological analysis.

During all the periods of the experiment, the animals were weighed daily, and the mean relative weight was calculated according to Zhang et al. [86]. This formula is defined as [(dn − d0)/d0], where d0 represents the mean animal original weight on the first day, and dn represents the mean animal weight after n days. Sodium dipyrone (200 mg/kg; Medley, Suzano, SP, Brazil) was administered twice every 24 h (4th and 5th days), according to veterinary recommendations for pain control.

### 4.5. Microbiological Analyses

The liver, kidney, and tongue were bisected into sagittal sections. The lung and spleen were bisected into coronal sections. Then, the left and lower halves of the organs were washed with sterile saline solution, weighed, and homogenized in 1 mL of sterile saline, followed by serial dilution. After, an aliquot of 100 µL of the homogenate and dilutions were spread on Sabouraud dextrose (SD) agar with chloramphenicol and incubated at 37 °C for 48 h. Subsequently, the number of colonies was counted, and the number of colony forming units per milligram of the organ was calculated (CFU/mg).

Furthermore, an aliquot of 100 µL of blood sample was added to 5 mL of peptone yeast extract glucose broth culture medium, followed by incubation at 37 °C for 72 h. Subsequently, an aliquot of 100 µL of broth cultivation was plated on SD agar with chloramphenicol and incubated at 37 °C for 48 h, and the number of colonies was determined.

### 4.6. Hematological Analyses

The blood samples obtained by cardiac puncture were placed in anticoagulant tubes containing ethylenediamine tetra acetic acid (EDTA) and were stored on ice until analysis. The complete hematologic analysis was performed using an automated methodology (EXIGO H-400). Quantitative analysis of red blood cells (RBCs), white blood cells (WBCs), and platelets was performed. The results were compared with hematological reference values [54].

### 4.7. Statistical Analysis

Data were statistically analyzed using the Graphpad prism version 8.0.1 (Graphpad Software Inc. San Diego, CA, USA). A normality test was performed to choose the suitable test according to the data variance. The body weights of the animals were compared using a Student’s paired *t* test. The data on the presence or absence of fungi in each organ in both groups were analyzed using Fisher’s exact test with aid of the software MatLab R2021a, Natick, MA, USA. The level of significance was set at 5% for all the tests.

## 5. Conclusions

In conclusion, cold atmospheric pressure plasma jets (CAPPJs) were safe and effective for reducing the dissemination of *C. albicans* to different organs in rats with chemotherapy-induced oral mucositis associated with candidiasis.

## Figures and Tables

**Table 1:** Erythrogram and leukogram results of the animals that underwent chemotherapy and were treated with cold atmospheric pressure plasma jets (CAPPJs) and non-treated controls. Results were obtained 24 and 72 h after the last session of treatment. Reference values [54] for normal rats are shown.

	Groups		
Blood Component	CAPPJs Treated	Non-Treated Control	Reference Values [54]
	24 h		
RBCs (10^6^/mm^3^)	5.98 ± 2.74	6.47 ± 3.21	6.6–9.0
WBCs (10^3^/mm^3^)	1.62 ± 1.04	1.70 ± 1.91	7.3–12.6
Platelets (mm^3^)	1159 ± 247.9	793.90 ± 564.65	840–1240
	72 h		
RBCs (10^6^/mm^3^)	4.78 ± 3.22	8.16 ± 2.19	6.6–9.0
WBCs (10^3^/mm^3^)	1.46 ± 1.31	1.77 ± 1.21	7.3–12.6
Platelets (mm^3^)	976.5 ± 349.9	1103.8 ± 203.1	840–1240

Legend: RBCs = red blood cells; WBCs = white blood cells (leukocytes). Reference hematologic values were reprinted/ adapted from Ref. [54], with permission John Wiley and Sons–Book. Copyright Wiley-Blackwell.

**Table 2:** Number of animals with positive yeast cultures in relation to the total (n/total) at the periods of 24 and 72 h after the second session of treatment with cold atmospheric pressure plasma jets (CAPPJs).

		Organs				
		Tongue	Lung	Liver	Spleen	Kidney
		24 h				
Number of animals with positive yeast cultures/total animals	CAPPJs	9/9	1/9	0/9	0/9	2/9
	Control	10/10	9/10	5/10	5/10	5/10
	*p* value	1.0000	* 0.0011	* 0.0325	* 0.0325	0.3498
		72 h				
	CAPPJs	7/7	0/7	0/7	0/7	0/7
	Control	9/9	5/9	4/9	3/9	3/9
	*p* value	1	* 0.0337	0.0885	0.2125	0.2125

Fisher’s exact test used, * *p* < 0.05.

## Data Availability

The data presented in this study are available from the corresponding author upon reasonable request.
